# Effect of monosodium glutamate on testicular tissue of paclitaxel-treated mice: An experimental study

**DOI:** 10.18502/ijrm.v17i10.5492

**Published:** 2019-11-28

**Authors:** Davoud Kianifard, Ali Ehsani, Parisa Zeinolabedini Daneshgar, Ghasem Akbari, Seyyed Maysam Mousavi Shoar4 Ph.D. Candidate

**Affiliations:** ^1^Department of Basic Sciences, Faculty of Veterinary Medicine, University of Tabriz, Tabriz, Iran.; ^2^Department of Food Science and Technology, Faculty of Nutrition and Food Sciences, Tabriz University of Medical Sciences, Tabriz, Iran.; ^3^Food and Drug Safety Research Center, Tabriz University of Medical Sciences, Tabriz, Iran.; ^4^Department of Basic Sciences, Faculty of Veterinary Medicine, Shahid Chamran University of Ahvaz, Ahvaz, Iran.

**Keywords:** Mice, Monosodium glutamate, Morphometry, Paclitaxel, Testicular tissue.

## Abstract

**Background:**

Paclitaxel (PTX), a chemotherapeutic agent, and monosodium glutamate (MSG) have oxidative effects on testicular tissue.

**Objective:**

In this study, the effects of MSG administration on the exacerbation of testicular tissue alterations related to PTX treatment were evaluated.

**Materials and Methods:**

MSG (30 & 60 mg/kg i.p.) was administrated to six groups (n = 8/each) of adult mice before or after PTX treatment: control, PTX-treated, MSG30 + PTX, MSG60 + PTX, PTX + MSG30, and PTX + MSG60. Following the euthanizing, the body weight measurement, pituitary–testicular axis hormonal analysis and serum lipid peroxidation index assessment was prepared, testicular histomorphometry (tubular diameter and germinal epithelium height), immunohistochemistry of *p53* was completed. Microscopic indices of spermatogenesis (tubular differentiation, spermiogenesis and repopulation indices) were studied.

**Results:**

Body weight was not changed significantly. The levels of testosterone (p = 0.0001), follicle stimulating hormone (p = 0.019), and luteinizing hormone (p = 0.08) were decreased while the level of lipid peroxidation index was increased (p = 0.208) in the treated groups. The histomorphometry indices (p < 0.0001 and p = 0.001, respectively), germ cells population (p < 0.05) and microscopic indices of spermatogenesis (p = 0.001, p = 0.005, p < 0.0001, respectively) were significantly reduced in all treated groups. The administration of MSG before PTX treatment induces more changes. The most positive reaction to *p53* was observed in MSG30 or 60 + PTX groups compared to other groups.

**Conclusion:**

The administration of MSG could intensify testicular tissue alterations related to PTX chemotherapy.

## 1. Introduction

Paclitaxel (PTX) is a mitotic inhibitor which is used as a chemotherapeutic agent for the treatment of solid tumors such as ovarian and prostatic cancers (1). The administration of PTX induced the alteration of spermatogenesis in rats (2). Monosodium glutamate (MSG) as a sodium salt of glutamic acid (amino acid) has toxic effects on various tissues of mammals (3, 4). Accordingly, it has been reported that some alterations of testicular tissue function such as hemorrhage and abnormal sperm production or morphology could be related to MSG consumption (4, 5). Moreover, environmental risk factors could affect the spermatogenesis process through induction of alterations in structure and function of germ cells (6). Due to the susceptibility of germinal epithelium of male reproductive system to chemotherapy agents and the lack of purposeful studies about the effects of the administration of MSG before or after chemotherapy, on the structural and functional alterations of testicular tissue induced by chemotherapy, this study was designed. Accordingly, given the importance of the nutrition and diet treatment in chemotherapy patients, the aim of this study was to evaluate the dose-dependent effects of the administration of MSG on the PTX-related testicular tissue alterations.

## 2. Materials and Methods

PTX (2 mg/kg b.w., Paclitaxel Stragen, STRAGEN PHARMA SA, Switzerland) was administered to mice intraperitoneally once per day for five consecutive days (1). MSG (30 & 60 mg/kg b.w., Sigma-Aldrich, St Louis, MO 63178, USA) was administrated intraperitoneally (7).

### Animal procedures

Forty eight adult male Syrian mice with mean body weight 30.43 ± 3.59 gr were divided randomly into six groups (n = 8/each) and placed in standard cages under 12-hr light/dark cycle. During the period of the experiment, the standard laboratory chow and water were available ad libitum to the animals.

### Experimental design

**Figure 1:**
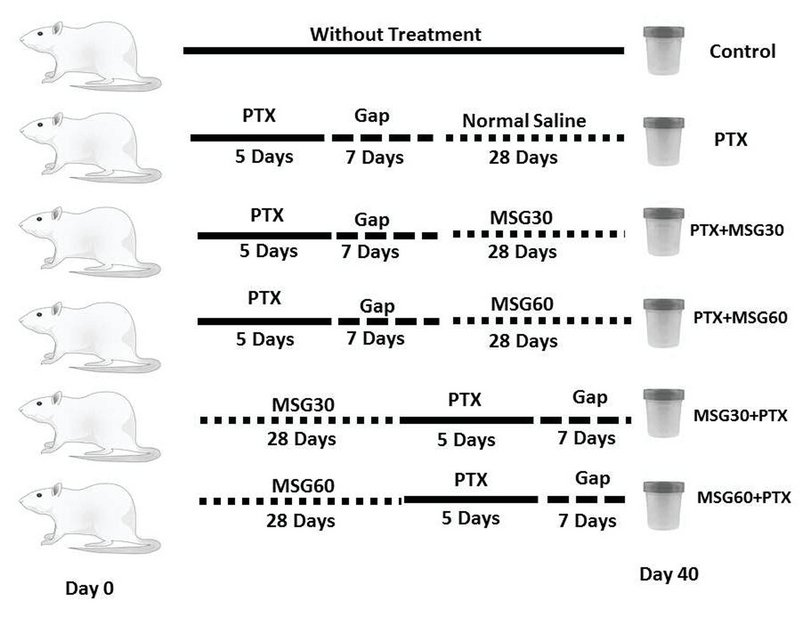
Flowchart of animal grouping and time schedule of the administration of MSG and PTX during the experimental study.

The animals were divided into six groups (Figure 1). 1)* Control* (normal and healthy mice that did not receive any type of treatment; 2) Paclitaxel treated (PTX) mice that received PTX for five consecutive days and after they were received normal saline for 28 days; 3) PTX+MSG30 group that received low-dose MSG daily for 28 days one week after the administration of PTX; 4) PTX+MSG60 group in which a high dose of MSG was administrated one week following the administration of PTX; 5) MSG30+PTX group that consisted of MSG30-treated animals that received MSG for 28 days before the administration of PTX; and 6) MSG60+PTX group that were treated similar to the previous group but with a high dose of MSG. The normal saline was injected to the animals of the control and PTX groups in an equivalent volume of PTX and MSG.

### Blood hormonal measurement and lipid peroxidation (MDA) assay 

At the end of the study, the animals were euthanized and the blood plasma was separated and kept at –80°C for the measurement of hormonal and malondialdehyde (MDA) levels. The standard ELISA method with commercial assay kits was prepared for quantitative analysis of FSH (Pishtazteb diagnostics, Iran), LH (Pishtazteb diagnostics, Iran), and testosterone levels (Monobind Inc. USA). The detection of MDA was carried out by commercial lipid peroxidation (MDA) assay kit based on MDA–TBA (thiobarbituric acid) complex formation.

### Tissue preparation and histological techniques 

Testicular tissues were fixed in 10% buffered formaldehyde solution (pH = 7.4). The paraffin embedded samples were cut and stained by Hematoxylin and Eosin for histomorphometrical observations.

### Histomorphometric analysis 

The height of germinal epithelium (GEH) and diameter of seminiferous tubules (STD) was investigated for morphometric analysis. Tissue micrographs were obtained by AmScope digital camera (AmScope MD 500) and processed by the image analysis software (AmScope 3.7). The samples were studied under ×100 magnifications.

### Evaluation of spermatogenesis in testicular tissue 

The assessment of spermatogenesis in testicular tissue was completed by the quantification of the number of seminiferous tubules with more than three layers of germinal cells derived from type-A spermatogonia (tubular differentiation index: TDI); the ratio of active spermatogonia to inactive cells (repopulation index: RI) and the ratio of seminiferous tubules with spermatozoids to the empty tubules (spermiogenesis index: SPI) (8).

### Immunohistochemistry (IHC) of *p53*

Paraffin-embedded tissue sections were prepared for IHC. Antigen retrieval was carried out on deparaffinised and rehydrated slides kept in 10 mM sodium citrate solution (pH 6.0) with temperature 95°C in a water bath for 40 min. Briefly, the activity of endogenous peroxidase was blocked with 0.3% H${}_{2}$O${}_{2}$. Tissue slides were washed with PBS (pH 7.2) and incubated with primary antibody (1:500) (Saint Johnes laboratory) at 4°C overnight. Sections were treated with horseradish peroxidase (HRP) conjugated secondary antibody in a humidified chamber for 1 hr. Diaminobenzidine (DAB) chromogen was added to tissue sections and incubated for 5 min. Tissue slides were dehydrated and cover slipped after hematoxylin counterstaining.

### Ethical consideration

All animal procedures used in this study were observed and approved by the Veterinary Research Ethics of the University of Tabriz standards for the care and use of laboratory animals (Code number: FVM.REC.1396.940).

### Statistical analysis

All results are expressed as mean ± SD. Statistical significance of differences between experimental groups was performed by one-way ANOVA followed by *Tukey's *multiple comparison test (GraphPad PRISMⓇ software version 5.04, Inc. USA). Differences with p < 0.05 were considered to be statistically significant.

## 3. Results

### Body weight

Table I shows the initial and final body weight in experimental groups. Accordingly, there was no significant difference between the experimental groups for initial and final body weight (p > 0.05). The lowest final body weight was observed in the MSG60 + PTX group. Similarly, the lowest body weight gain was observed in the MSG60 + PTX and MSG30 + PTX groups. The administration of MSG before the treatment of animals with PTX led to lower weight gain in comparison to the other groups.

### Hormonal assay and serum MDA

Table II shows the changes in the hormones of the pituitary–testicular axis and serum MDA levels. Accordingly, the administration of PTX and MSG led to a decrease in the blood levels of FSH, LH, and testosterone. The blood concentration of testosterone was reduced significantly in all the treated groups in comparison to the control group (p = 0.0001). The most reduction in FSH level was observed in the MSG30 + PTX and MSG60 + PTX groups (p = 0.019), while the blood concentration of LH was significantly decreased only in the MSG60 + PTX group (p = 0.083). The mean of blood MDA levels was increased in all the treated groups in comparison to the control group (p > 0.208). In this regard, the administration of MSG with PTX induced more elevation of blood MDA levels compared to the untreated PTX group. Moreover, the administration of MSG before an injection of PTX led to more increment of MDA levels in comparison to the other groups that received MSG after the injection of PTX.

### Testicular histomorphometry and the population of germinal epithelium

**Figure 2:**
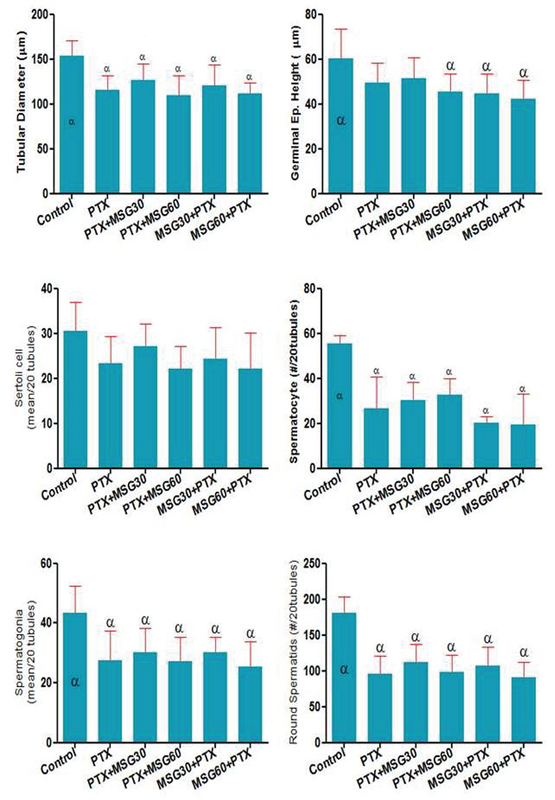
Testicular tissue histomorphometry and the population of germinal epithelium in experimental groups. (${}^{\alpha }$Significantly different in comparison to the control group. Data are presented as mean ± SD.)

The use of PTX led to decrease of tubular diameter and the GEH in comparison to the control group (Figure 2). Simultaneous administration of PTX and MSG led to decrease of morphometric indices. The mean of tubular diameter was reduced in all experimental groups compared to the control group (p < 0.0001). Moreover, the GEH was decreased in the experimental groups. This decrease was significant in (PTX + MSG60), (MSG30 + PTX), and (MSG60 + PTX) groups (p = 0.0013). Accordingly, these decrements were dose-dependent and more visible in the MSG + PTX groups in comparison to the PTX + MSG groups. The mean of the population of spermatogonia, spermatocytes, and spermatids was reduced significantly in all the treated groups in comparison to the control group (p < 0.0001, p = 0.0004, p < 0.0001, respectively). The population of Sertoli cells was reduced in all the treated groups in comparison to the control animals (p = 0.105).

### Microscopic indices of spermatogenesis

The mean of microscopic indices of spermatogenesis (TDI, SPI, and RI) were reduced in all the treated groups compared to the control group (Table III). The administration of PTX led to a significant decrease of these indices in comparison to the control group (p < 0.05). Accordingly, the lowest degree of these indices was recorded in the MSG60 + PTX group.

### Histology of testicular tissue

**Figure 3:**
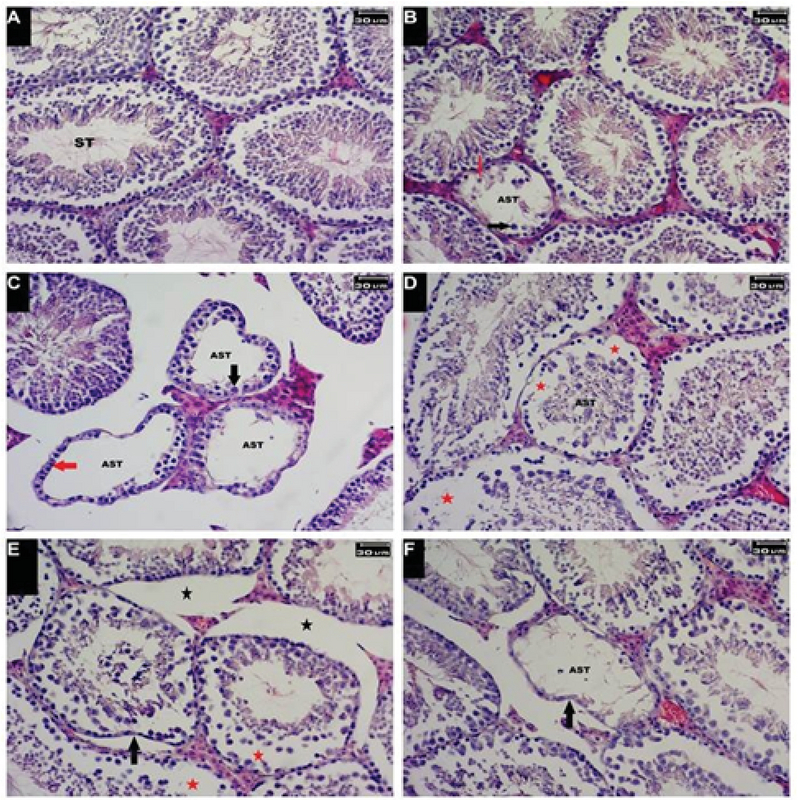
Histology of testicular tissue in experimental groups. (A) Control group. Seminiferous tubules with normal architecture; (B) PTX-treated group. Tubular atrophy with a decrease of the cellular population and nuclear displacement of Sertoli cells (red arrow). Decrease in the population of spermatocytes (black arrow) is visible; (C) PTX + MSG60 group. Tubular atrophy with a decrease in the population of spermatocytes (black arrow). Some tubules were observed without spermatogenic cell line (red arrow); (D) PTX + MSG30 group. Decrease of cellular population (red asterisks) with interstitial edema; (E) MSG30 + PTX group. Increase in connective tissue (black asterisks), tubular atrophy (black arrow) with a decline in the cell population (red asterisks) is visible; (F) MSG60 + PTX group. Tubular atrophy with severe depletion in the cell population was observed. (AST: Affected seminiferous tubule; ST: seminiferous tubule. H & E staining; Magnification: ×400.)

Figure 3 shows the histology of testicular tissue in the experimental groups. Accordingly, in the PTX-treated group, cellular and structural alterations such as tubular atrophy, interstitial edema, and depletion in cell population were observed (Figure 3B). These changes were detected in groups treated with PTX and MSG simultaneously (Figure 3 CF). These structural changes were observed more in the MSG + PTX groups (Figures 3E and 3F) in comparison to the PTX + MSG groups (Figures 3C and 3D).

### Immunohistochemistry of *p53*

**Figure 4:**
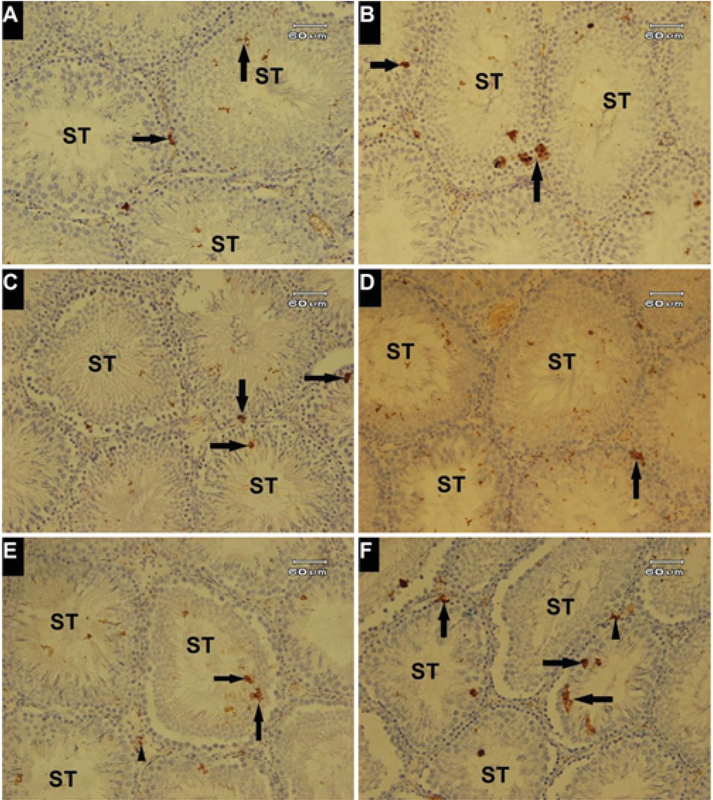
Expression of *p53* protein in testicular tissue. Immunohistochemical study. (A) Faint positive reaction observed (Arrows) in seminiferous tubules (ST); (B) positive reaction to *p53* expression (Arrows) in PTX-treated group; (C&D) *p53* positive reaction in germinal epithelium (Arrows) in the PTX + MSG30 and PTX + MSG60 groups; (E&F) Increment of positive reaction was observed in germinal epithelium (Arrows) with positive reactivity of interstitial connective tissue (Arrowheads) in the MSG30 + PTX and MSG60 + PTX groups. Magnification ×200.

The study of testicular seminiferous tubules for the detection of the expression of *p53* showed a different reactivity (Figure 4). Immunohistochemical study of testicular tissue of the control group revealed faint immunostaining in the control group (Figure 4A). The positive reaction areas were increased in the treated groups in comparison to the control group. In this regard, the expression of *p53* was observed in the germinal epithelium of the seminiferous tubules. Moreover, the positive reaction areas were observed in more degrees in the MSG30 + PTX and MSG60 + PTX groups (Figures 4E and 4F) compared to the PTX + MSG30 and PTX + MSG60 groups (Figure 4C and 4D). Also, the administration of MSG led to an increase of *p53* expression in the MSG-treated animals in comparison to the PTX-treated group. High dose of MSG induces more positive reaction areas compared to a low dose of MSG.

**Table 1:** Mean of initial and final body weight in experimental groups


	**Initial body weight (gr)**	**Final body weight (gr)**	**Weight gain (gr)**
Control	27.80 ± 6.78	36.00 ± 3.65	8.2
PTX	31.60 ± 1.67	36.00 ± 2.00	4.4
PTX + MSG30	28.40 ± 2.96	34.40 ± 2.88	6.0
PTX + MSG60	31.20 ± 2.68	35.60 ± 2.96	4.4
MSG30 + PTX	32.80 ± 1.09	35.00 ± 3.28	2.2
MSG60 + PTX	30.80 ± 1.78	34.00 ± 1.41	3.2
P-value	0.198	0.816	
Data are presented as Mean ± SD.			
PTX: Paclitaxel; MSG: Monosodium glutamate

**Table 2:** Blood hormonal and malondialdehyde levels in experimental groups


	**FSH (IU/L)**	**LH (IU/L)**	**Testosterone (ng/ml)**	**Serum MDA nmol/mg protein**
Control	0.28 ± 0.02	0.24 ± 0.02	1.07 ± 0.13	0.78 ± 0.13
PTX	0.25 ± 0.04	0.19 ± 0.05	0.84 ± 0.13${}^{\alpha }$	0.95 ± 0.22
PTX + MSG30	0.25 ± 0.03	0.20 ± 0.03	0.76 ± 0.11${}^{\alpha }$	0.97 ± 0.19
PTX + MSG60	0.23 ± 0.04	0.21 ± 0.04	0.83 ± 0.12${}^{\alpha }$	0.94 ± 0.26
MSG30 + PTX	0.22 ± 0.04${}^{\alpha }$	0.20 ± 0.03	0.82 ± 0.14${}^{\alpha }$	1.00 ± 0.20
MSG60 + PTX	0.22 ± 0.03${}^{\alpha }$	0.18 ± 0.02${}^{\alpha }$	0.79 ± 0.04${}^{\alpha }$	1.15 ± 0.23
P-value	0.019	0.083	0.0001	> 0.208
Data are presented as Mean ± SD. ${}^{\alpha }$ Significant different in comparison to the control group.			
PTX: Paclitaxel; MSG: Monosodium glutamate; FSH: Follicle stimulating hormone; LH: Luteinizing hormone; MDA: Malondialdehyde

**Table 3:** Mean of microscopic indices of spermatogenesis in experimental groups


	**TDI (%)**	**SPI (%)**	**RI (%)**
Control	69.70 ± 12.26	67.70 ± 10.03	84.14 ± 9.26
PTX	50.90 ± 15.26${}^{\alpha }$	49.10 ± 9.63${}^{\alpha }$	63.00 ± 10.49${}^{\alpha }$
PTX + MSG30	54.10 ± 11.72	53.60 ± 10.32	55.86 ± 8.29${}^{\alpha }$
PTX + MSG60	48.0 ± 11.10${}^{\alpha }$	51.60 ± 12.83${}^{\alpha }$	58.14 ± 8.03${}^{\alpha }$
MSG30 + PTX	52.50 ± 12.20${}^{\alpha }$	54.30 ± 13.99	56.14 ± 11.44${}^{\alpha }$
MSG60 + PTX	45.50 ± 12.99${}^{\alpha }$	46.40 ± 14.91${}^{\alpha }$	51.86 ± 5.78${}^{\alpha }$
P-value	0.001	0.005	< 0.0001
Data are presented as Mean ± SD. ${}^{\alpha }$ Significant different in comparison to control group.			
PTX: Paclitaxel; MSG: Monosodium glutamate; TDI: Tubular differentiation index; SPI: Spermiogenesis index; RI: Repopulation index

## 4. Discussion

In this study, the effect of MSG on testicular tissue of paclitaxel-treated mice was evaluated. The results obtained from this study showed that the administration of MSG during the period of chemotherapy (paclitaxel) can exacerbate the testicular tissue alterations related to PTX treatment. The comparison of the results obtained from the measurement of various cellular and histological parameters revealed that there is a difference in observed changes between formerly administration of MSG and consequent administration of MSG. Additionally, one of the prominent findings in this study was that the administration of MSG before treatment of animals with PTX could induce more changes in the structure and function of testicular tissue compared to other groups which received the MSG subsequent of PTX treatment. Various environmental factors could induce different fertility alterations in male reproductive system (9). The toxicity of MSG on the structure and function of testicular tissue of human and experimental animals has been reported (10, 11). In this regard, it has been stated that the structural alteration of testicular tissue related to the administration of MSG is mediated by oxidative damage (12). Furthermore, some studies demonstrated certain abnormalities as male infertility, obesity and hypogonadism associated to MSG consumption (13, 14). Some tissues and organs of rats as well as testicular tissue have the glutamate receptors (15, 16). Oxidative stress is involved in decline of completion of functional maturation and capacitation of spermatozoa due to decrement of glutathione levels in male germ cells (17, 18).

One of the possible mechanisms of testicular tissue alterations in MSG-treated animals could be related to oxidative damages of the germ cells following the administration of MSG. The present study showed that the blood MDA contents were enhanced in all experimental groups compared to the control animals. This increase of serum MDA levels were observed in the groups treated with both PTX and MSG in comparison to the PTX-treated mice. Accordingly, our results showed that the administration of MSG prior to the PTX treatment induces more elevation in blood levels of MDA in dose-dependent manner. Along with the aforementioned results, the most histomorphometrical and histochemical alterations were observed in the MSG + PTX groups compared to the PTX + MSG groups. These results confirm that the administration of MSG through the induction of oxidative stress could involve in the intensity of cellular and tissue alterations related to the PTX treatment. In this regard, it has been reported that the PTX has a direct effect on the formation of free radicals by inducing intracellular oxidative stress (19-21). However, in our study the tissue MDA levels were not measured.

In this study, the negative impact of PTX and MSG on the reproductive system of male mice was considered by noteworthy histological changes such as decline of spermatogenesis indices, decrease in tubular morphometry, disarrangement, and depletion of germinal epithelium. In MSG-treated animals, the reduction of the conversion of spermatogonia to primary spermatocytes leads to diminished TDI and SPI rate. As well, the increase of the population of inactive spermatogonia reduces RI index which induces a decline in the population of primary spermatocytes and the reduction in the production of spermatozoids consequently. Accordingly, the results of this study showed that the administration of MSG before or following of PTX treatment induce a reduction of RI in dose-dependent manner. In some studies, it has been stated that the RI decreases in the PTX-treated rats (22). This reduction of RI could be related to the antiproliferative properties of PTX. However, the results of our study revealed that the animals which received MSG prior to PTX had a lower RI percentage in comparison to the other animals that received MSG after the PTX treatment. According to these results, we can suggest that the administration of MSG could be effective in exacerbation of tissue damages induced by PTX through cytotoxic processes such as excessive ROS production and induction of oxidative stress.

The results of the present study showed that the treatment with PTX reduces weight gain process. The weight loss has not been reported in the PTX-administrated adult rats (23). Conversely, an increase of body weight has been reported in the administration of MSG (24). Our previous study revealed that the administration of MSG at high dose induces more weight gain in comparison to its lower dose (9). It seems, the effects of MSG on the weight gain process can occur in a dose-dependent manner. Once 40 days of study, the rate of weight gain in the animals treated with PTX was lower in comparison to the control group. These changes of body weight could be related to PTX-induced anorexia and subsequently low food intake. Decrease in weight gain that occurs in all PTX-treated animals could be related to the PTX central effects or pathologic conditions of gastrointestinal system that lead to a decrease of the activities of digestive system. In this regard, peripheral neuropathy is the most common side effects of PTX administration (25). However, it does not seem that the PTX-induced neuropathy could be the main factor in the process of lower weight gain. So, according to the previous studies (22), it appears that the interruption of the motility of digestive system associated to decreased neuronal activities of gastrointestinal system related to PTX treatment could be effective in lower weight gain in the PTX-treated animals. These changes in weight gain were more visible when MSG was administrated before the PTX treatment.

It has been reported that the administration of MSG induces a reduction of serum LH and testosterone levels (26). Accordingly, the administration of MSG to adult mice induces cellular damage of hypothalamic neurons in *arcuate* and *preoptic* nucleuses (11, 27, 28). As a result, the dysfunction of the hypothalamic–pituitary–gonadal axis could induce some alterations in the testicular function (29). In this study, the administration of PTX had no significant effect on the pituitary gonadotropins but the blood levels of testosterone reduced significantly. This reduction of testosterone levels could be related to the negative effects of PTX on the Leydig cells' division which may lead to a decline in the population of these cells as a main source of testosterone production. This factor could be one of the mechanisms involved in the reduction of male fertility following the chemotherapy.

PTX is a mitotic inhibitor chemotherapeutic agent used for the treatment of solid tumors such as ovarian and prostatic cancers (25). The binding of PTX to β-tubulin of microtubules leads to an increase in microtubule polymerization (30). It has been demonstrated that the administration of PTX influences actively dividing and post-meiotic cells which induce some alterations in spermatogenesis by damaging spermatogonia in the delayed periods (31). In this study, the results obtained from testicular morphometry and cell population revealed an alteration in the structure of seminiferous tubules. In this regard, the depletion of tubular cell population, especially decrease in the number of dividing cells, induces decrement in spermatogenic indices which accomplished with structural changes in seminiferous tubules. These changes were observed in the PTX and other groups that received MSG before or after the PTX treatment. Accordingly, it seems that the treatment with PTX following the administration of MSG induces these aforementioned changes in more degrees.

In this study, for the estimation of DNA damage in germinal cell lineage, the expression of *p53* protein was evaluated. According to our results, the most positive reactions were observed in the testicular tissue of animals that had received MSG prior to the PTX treatment. These results indicate that the administration of MSG before the PTX treatment could induce more DNA damage and subsequently cell death. Programmed cell death (apoptosis) is a normal regulatory process in testicular tissue and occurs in various phases in germinal epithelium (32, 33). The changes in nuclear DNA of testicular germ cell lineage could involve in spermatogenesis failure (34). Furthermore, cellular stress such as severe DNA damage induced by exogenous factors could generate the upregulation of *p53* expression that can activate the initiation of apoptosis or repair pathways in the cell (35). Likewise, the over expression of *p53* proteins could induce the abnormal cell death and consequently decrease in the cell population and subsequently the reduction in sperms quality and quantity (32, 33, 36). According to the increase of *p53* positive areas in testicular samples of PTX + MSG or MSG + PTX groups, we can propose that the administration of MSG to PTX treated animals could accelerate the PTX-associated germinal epithelium apoptosis through upregulation of *p53*, dose-dependently.

## 5. Conclusion 

According to the results of this study, it has been concluded that the administration of MSG at the time of PTX chemotherapy could involve in intensifying of structural and functional changes of testicular tissue which may lead to several degrees of fertility alterations. Since the mechanism of the activity of PTX differs from the MSG, it appears that the raise of tissue oxidative stress related to MSG administration could induce DNA damage which leads to a decline in the population of germ cells. Finally, the reduction in the cell division induced by PTX with negative tissue-damaging effects of MSG and the alteration of the pituitary–testicular axis hormones could promote further damage of testicular tissue. It seems that the administration of MSG prior to PTX treatment has more negative effects on reproductive system in comparison to the administration of MSG after the paclitaxel treatment.

##  Conflicts of Interest

There is no conflict of interests in this study.
